# Menopausal status is associated with a high risk for residual disease after cervical conization with positive margins

**DOI:** 10.1371/journal.pone.0217562

**Published:** 2019-06-04

**Authors:** João Paolo Bilibio, Heleusa Ione Monego, Márcia Luiza Appel Binda, Ricardo dos Reis

**Affiliations:** 1 Postdoctoral Program of the Programa de Pós Graduação de Ciências Médicas da Universidade Federal do Rio Grande do Sul, Porto Alegre, Rio Grande do Sul, Brazil; 2 Department of Obstetrics and Gynecology, Universidade Federal do Pará, Belém, Pará, Brazil; 3 Department of Obstetrics and Gynecology, Universidade Federal do Rio Grande do Sul, Hospital de Clínicas de Porto Alegre, Porto Alegre, Rio Grande do Sul, Brazil; 4 Gynecologic Oncology Department, Barretos Cancer Hospital, Barretos, São Paulo, Brazil; Rudjer Boskovic Institute, CROATIA

## Abstract

**Background:**

We aimed to determine demographic and clinicopathological predictors for residual disease in women with cervical intraepithelial neoplasia (CIN 2/3) with endocervical cone margin involvement.

**Methods and findings:**

A cross-sectional study was conducted. The eligible patients were women who underwent hysterectomy as a treatment option after having a positive endocervical margin for CIN 2/3 in cervix conization specimens from 2000 to 2015. The patients were divided into two groups based on the persistence of CIN 2/3 and absence of CIN 2/3 in hysterectomy specimens. Demographic, clinical and histology information were collected in both groups. A total of 80 patients were eligible for the study; 37 (46.3%) had no persistence of CIN 2/3 and 43 (53.7%) had persistence of CIN 2/3 in the hysterectomy specimens. Demographic, clinical, and cone specimen characteristics, and a visible squamocolumnar junction and type of conization were analyzed as possible risk factors for the presence of residual lesions at hysterectomy, and none of these variables were associated with residual disease. Menopausal status was strongly associated with a high risk of persistent residual disease 81.2% (OR 4.9, CI 1.27–18.9), *P* = 0.014. In the multivariate analysis, only a menopausal status (*P =* 0.04) was associated with a high risk of persistent lesions.

**Conclusion:**

This analysis found that menopausal status exhibited an important association with persistent residual disease. Menopausal women with endocervical margin involvement exhibit a greater than 80% risk of persistent lesions.

## Introduction

Cervical cancer is the second most common malignant neoplasm in women worldwide. High-grade cervical intraepithelial neoplasia (CIN 2/3) is associated with a high risk of developing cervical cancer and is typically treated with conization (cold knife or loop electrosurgical excision procedure (LEEP)). The status of the endocervical cone margin is associated with a major risk for persistence/recurrence of this disease [1–2]. It is estimated that the rate of recurrence two years after treatment in patients without compromised margins is approximately 4 to 18% with an average of 8% [3]. However, in patients with positive margins, this risk of recurrence is typically higher, and some studies report rates of approximately 52% [1–4].

The ideal follow-up for patients with positive margin involvement after conization is controversial among experts. Some patients who have positive margins will not present residual disease, whereas another portion will still have residual disease in subsequent resection specimens [2, 5–7]. Thus, some experts suggest clinical follow-up with colposcopy and cytology, whereas others indicate surgical treatment, such as reconization or hysterectomy. The decision is often made based on age, reproductive desire, availability of appropriate follow-up, or presence of other concomitant gynecological problems that can be an indication of hysterectomy, including fibroids, uterine prolapse, or other associated pathologies, such as endometriosis [7].

If the choice of treatment is new conization or hysterectomy, a portion of women will undergo these procedures unnecessarily because they do not have a residual lesion. This unnecessary surgery increases the risks and complications and highly affects their gestational future, i.e., premature preterm labor, premature rupture of membranes or the need for a surrogate uterus in patients who undergo hysterectomy [2, 5–7]. Alternatively, if surgery is not performed, there is a risk of inadequately treating a large number of patients who have CIN 2/3 with a risk of malignancy. The estimated prevalence of positive margins after conization could be greater than 30% [2, 7]; thus, many researchers are searching for risk factors that may be associated with persistence/recurrence of CIN 2/3 that could facilitate the identification of the correct treatment for each patient.

The identification of these predictors is fundamental to determine the correct follow-up in patients with positive endocervical margins. The aim of this study was to determine the demographic and clinicopathological predictors for residual disease in hysterectomy specimens in women after conization for CIN 2/3 with endocervical cone margin involvement.

## Methods

This cross-sectional study was conducted in patients with CIN 2/3 who had positive endocervical margin involvement after conization and underwent a total hysterectomy. The study was conducted at the Gynecologic Oncology Service of the Hospital de Clínicas de Porto Alegre (HCPA), University Hospital, Department of Obstetrics and Gynecology, from 2000 to 2015. The study was approved by the Ethics Committee of the Comitê Nacional de Ética em Pesquisa and by the Comitê de Ética em Pesquisa do Hospital de Clínicas de Porto Alegre (HCPA) (institutional review board equivalent). The data were collected through the electronic medical records of the institution (HCPA) while preserving the patients' anonymity and the research ethics committee waived the requirement for informed consent because the study used previously stored data.

Eligible patients for this study included women who underwent hysterectomy as a treatment option after having a positive endocervical margin for CIN 2/3 in cervix cone specimens after treatment with a cold knife or LEEP. In total, 128 patients were selected for inclusion in this study. Of these, 48 cases were excluded based on the following exclusion criteria: patients underwent hysterectomy for another indication, such as myomectomy, abnormal uterine bleeding, cervical stenosis or another indication; presence of a disease that could affect the immune system, such as HIV or an immunological disease; chronic use of corticoids or immunosuppressive drugs; chemotherapy or radiotherapy; and patients with low-grade dysplasia at conization. Eighty patients were eligible for this study. After initial conization, the selected patients had returned for a consultation and were informed that the endocervical margin was compromised. It was explained that they would need a new procedure, either a new conization or a hysterectomy if they did not want to preserve the fertility potential. All these patients underwent total hysterectomy for positive endocervical margin involvement of CIN 2/3 after conization because they had already completed their fertility and follow up with a specialist was difficult due to their geographic location. The hysterectomy specimens of these patients were divided into two groups: women with residual disease (residual disease group) and women with no residual disease (no residual disease group).

Data with a normal distribution were analyzed using Student’s t-test or ANOVA for independent samples and Levene’s test for equality of variances. The chi-square test was used for categorical variables. A logistic regression model was used to determine the association between various patient characteristics and residual disease in the multivariate analysis. The threshold for statistical significance was 5%. Statistical tests were performed using the Statistical Package for the Social Sciences 20 (SPSS Inc., Chicago, IL, USA). Moreover, we performed an analysis to verify the power calculation. Our results showed sufficient power with a Fisher's test of 87.8% and a Mid-P test of 91.6% using our data. Therefore, our findings are robust and valuable, considering adequate power greater than 80%.

## Results

Hysterectomy specimens from a total of 80 patients were analyzed; 37 (46.3%) had no persistence of CIN 2/3 (no residual disease group) and 43 (53.7%) had persistence of CIN 2/3 (residual disease group). The analysis of demographic and clinical characteristics as possible risk factors for the presence of residual lesions at hysterectomy (age, parity, previous cesarean, previous abortion, race, menstrual cycles, body mass index) is presented in Table 1. No statistical difference was observed between the no residual disease group and residual disease group.

**Table 1:** Analysis of the demographic and clinical characteristics as possible risk factors for the presence of residual disease in the hysterectomy specimens of women after conization for CIN 2/3 with endocervical cone margin involvement (mean±SD).

Parameter	No residual disease group (n = 37)	Residual disease group (n = 43)	*P-*value^a^
Age (years)	42.3±11.3	47.3±13.3	0.075
Parity (n)	3.9±2.4	3.6±2.9	0.629
Caesarean (n)	0.5±0.7	0.5±0.8	0.668
Abortion (n)	0.3±0.9	0.3±0.5	0.911
Race % (n)			
White	48.6% (18)	51.2% (22)	
Latin American	48.6% (18)	44.2% (19)	
African American	2.8% (1)	4.6% (2)	0.856^c^
Menstrual cycles (days)^d^	29.9±2.1	29.8±2.0	0.960
BMI^b^ (kg/m^2^)	24.2±3.0	24.2±2.7	0.760

^a^ Student’s t-test or chi-square test.

^b^ Body mass index.

^c^ Chi-square test or Fisher’s exact test.

^d^ Only 63 women were included in this analysis, since the remainder were already in menopause

Analysis of cone surgical characteristics as possible risk factors for the presence of residual lesions at hysterectomy (cone height, cone width, conization time until hysterectomy) is presented in Table 2. None of these variables were associated with residual disease.

**Table 2:** Analysis of cone surgical characteristics as possible risk factors for the presence of residual disease in the hysterectomy specimens of women after conization for CIN 2/3 with endocervical cone margin involvement (mean±SD).

Parameter	No residual disease group (n = 37)	Residual disease group (n = 43)	*P-*value^a^
Transverse cone width (mm)	22.6±6.1	23.0±6.7	0.791
Anteroposterior cone width (mm)	17.0±6.2	15.3±5.2	0.268
Cone depth (mm)	20.0±6.1	21.9±9.5	0.317
Conization time until hysterectomy (month)	4.1±4.3	7.4±13.3	0.163

^a^ Student’s t-test.

The analysis of menopausal status, a visible squamocolumnar junction (SCJ) and type of conization (LEEP or cold knife) as possible risk factors for the presence of residual lesions at hysterectomy is presented in Table 3. We found that menopausal status was strongly associated with a high risk of persistence of residual disease 81.2% (OR 4.9, CI 1.27–18.9), *P* = 0.014.

**Table 3:** Analysis of menopausal status, squamocolumnar junction (SCJ) status and surgical technique as possible risk factors for the presence of residual disease in the hysterectomy specimens of women after conization for CIN 2/3 with endocervical cone margin involvement.

Parameter	No residual disease group	Residual disease group	*P-*value^a^	OR (95% CI)	*P-*value^b^
Premenopausal status	(34) 53.1%	(30) 46.9%			
Postmenopausal status	(3) 18.8%	(13) 81.2%	0.014	4.9 (1.27–18.9)	0.020
SCJ visible	(30) 52.6%	(27) 47.4%			
SCJ not visible	(7) 30.4%	(16) 69.6%	0.072	2.5 (0.90–7.10)	0.075
Cold knife	(18) 39.1%	(28) 60.9%			
Electrosurgical knife	(19) 55.9%	(15) 44.1%	0.137	0.5 (0.20–1.24)	0.139

^a^Chi-square test

^b^ P-value of the risk estimate (odds ratio)

The logistic regression analysis for the presence of residual lesions at hysterectomy (dependent variable: residual disease, independent variables: menopausal status and cone depth) is presented in Table 4. Menopausal status was associated with residual disease (*P =* 0.04), whereas cone depth was not (P = 0.550).

**Table 4:** Logistic regression analysis for the presence of residual lesions at hysterectomy (dependent variable: residual disease, independent variables: menopausal status and cone depth).

	B	Sig.	Exp (B)	95% CI for Exp (B)
Menopausal status	1.419	0,044	4.132	1.042–16.380
Cone depth	0.018	0.550	1.018	0.959–1.081
Constant	-0,474	0.485	0.625	

## Discussion

In this study, approximately 53% of women who underwent hysterectomy for endocervical cone margin involvement of CIN 2/3 after conization exhibited persistent disease in hysterectomy specimens. This finding demonstrates a high prevalence of residual disease if endocervical margin involvement was noted after conization. This result is similar to findings of other studies that reported persistence rates of approximately 45–55% in this situation [1, 4, 8]. In this study, menopausal status was strongly associated with a high risk of persistent disease: 81.2% in menopausal women versus 46.9% in nonmenopausal women. Other studies have previously demonstrated menopausal status as a risk factor for persistent lesions. Xiang et al evaluated the incidences of margin involvement after electrosurgical knife and there were three factors associated with positive margins: age greater than 50 years (odds ratio 3.0), postmenopausal status (odds ratio, 3.1) and microinvasive disease (OR, 2.7) [9]. Similar results were found in other studies that also evaluated factors associated with positive cone margin status after electrosurgical knife and also concluded that increased age are associated with persistent/recurrent disease [2, 10]. These three studies described above only included patients who performed electrosurgical knife, different from ours, that we include patients who performed electrosurgical and cold knife.

The main hypothesis for this increased risk of persistent lesions in menopausal women (approximately 80%) is hypoestrogenism [11–12]. Estradiol (E2) and progesterone secreted during the menstrual cycle act both directly and indirectly on epithelial cells, fibroblasts and immune cells in the reproductive tract to modify immune function in a manner that is unique to specific sites throughout the female reproductive tract [13]. Estrogen is related to humoral immunity, and a decline in its production attenuates the immune response by predisposing individuals to microbial invasion and infection. Thus, reduced estrogen in postmenopausal women decreases the number of cells secreting IFN-γ and TNF-α, contributing to the reduction of immunological reactivity. Hypoestrogenism favors the release of proinflammatory cytokines, whereas estrogen exerts an opposite effect by negatively influencing the cervical epithelium, favoring the onset of dysplasia, increasing lesion growth, and reducing the effect of cauterization and healing at the positive margin of the lesion [11–13]. This notion explains why menopausal status could be associated with the risk for residual disease. Thus, we believe that menopausal women require a different follow-up protocol than premenopausal patients. Menopausal patients should undergo a more rigorous postoperative follow-up, indicating immediate reconization or hysterectomy for definitive treatment.

Other reasons that might explain this increased risk in menopausal women were refuted by the results of our study. During menopause, the SCJ is located deeper in the cervical canal, causing partial withdrawal of the lesion with conization [14]. Thus, lesions that enter into the endocervical canal may not be completely visible, leading to a risk of residual lesion; however, we did not identify this factor as a possible cause of lesion persistence. Thus, consistent with previous studies, we verified that lesion persistence does not seem to be related to lesion visibility [15–19].

Another factor that could influence lesion recurrence is the depth of the cone specimen. Menopausal patients may have deeper lesions because the SCJ is not visible. A shallow cone results in incomplete resection of the lesion. Previous studies have reported that a cone depth less than 10 mm significantly increases the rate of residual disease, whereas a cone depth greater than 18–20 mm excludes this possibility [20–21]. Other studies have evaluated (through ROC curve analyses) the best cutoff point for cone depth to decrease the risk of residual disease. Bay found three ideal cutoff points for cone depth according to patient age and type of CIN: in women under 50 years of age, the cut-off value was achieved at 1.8 cm cone depth (area under the curve (AUC) 0.64, sensitivity 0.86); for a subset of CIN II patients aged less than 50 years, the cut-off value was 1.2 cm (AUC 0.75, sensitivity 0.90); in women <40 years of age, the cut-off value was 1.8 cm (AUC 0.60, sensitivity 0.88); and in a subset of CIN II, the cut-off value was 0.9 cm (AUC 0.87, sensitivity 0.83 (Bae 2013). Papoutsis showed that women are at risk for positive margins after large loop excision of the transformation zone (LLETZ) when the cone length is less than 10 mm [22]. Comparing these studies with our results (mean depth cone: No residual disease group: 20.0 mm versus Residual disease group: 21.9 mm), we can conclude that the depth of the cone specimen was technically adequate to prevent residual disease in our patients. Furthermore, in the comparison between groups and in logistic regression analysis we did not identify an association between cone depth and persistence of lesion.

Other researchers revealed an association between potentially false results of positive margins after conization and the type of technique used (cold knife or LEEP). The effect of cauterization of the bloated area due to the LEEP could cause a local inflammatory reaction with the release of innumerable immunological mediators and the attraction of lymphocytes and other immunological cells, thus reducing the risk of injury persistence [23]. However, this association was not observed in our study and other published studies [24].

The strengths of our study include the relatively large number of patients and the fact that we exclusively included patients with endocervical margin involvement, given the lack of consensus regarding adequate treatment for these patients. In addition, this is a retrospective study, representing the experience of a single center. Furthermore, all patients that participated in this study and met the inclusion and exclusion criteria, the percentage of missing data and cases lost to follow-up were minimal.

The results of our study are of great importance for clinical practice. Currently, the Brazilian Guidelines for the Screening of Uterine Cervical Cancer of the National Cancer Institute 2016 (INCA) for the follow-up of patients with compromised endocervical margins suggest cytopathology and colposcopy every six months during the first two years and thereafter annually until the fifth year [25]. However, given a persistent lesion rate of approximately 80% in menopausal patients, this protocol should be revised in menopausal patients. In fact, it would be prudent for these patients to undergo a new procedure, such as reconization for patients who wish to become pregnant or total hysterectomy. It seems reckless that patients with an 80% increased risk of persistent injury are only clinically monitored.

## Conclusion

To summarize, in the present study, we identified that menopausal status exhibited an important association with persistent residual disease. These results indicate that repeat cervical conization rather than cytopathology and colposcopy during follow-up is viable for patients with endocervical margin involvement with CIN 2/3 after conization. Besides that, we have to remember that the squamocolumnar junction is not visible in the majority of menopausal patients after conization. Notably, conization specimens with endocervical margin involvement exhibit a persistence rate greater than 80% in menopausal women. Patients without these characteristics can undergo more conservative follow-up treatment.

## Supporting information

S1 Checklist STROBE Statement—Checklist of items that should be included in reports of observational studies. (DOC)
